# Art of medicine, art as medicine, and art for medical education

**DOI:** 10.36834/cmej.70298

**Published:** 2020-12-07

**Authors:** Patricia Lynn Dobkin

**Affiliations:** 1 Department of Medicine, McGill University, Quebec, Canada

How often have we heard the phrase “the art and science of medicine?” We may consider how medical practice is, in part, “art” i.e. an aspect of one’s practice of medicine, how it can promote healing, as well as how art can enhance the formation of future doctors. Throughout this commentary I base my proposals on the notion that art can impact trainees and physicians directly and that they may, in turn, promote healing in patients either indirectly by being a “whole person-doctor” or directly by integrating various forms of art therapy into their treatment. At McGill Programs in Whole Person Care our approach is based on the concept that it takes the “whole person” of the clinician to treat the “whole person-patient.” (for the mission statement see: www.mcgill.ca/wholepersoncare)

Let us start with ***the art of medical practice***. The art of medical practice refers to a clinician’s way of being when interacting with patients and families. How doctors, nurses, and allied health care professionals (HCPs) diagnose, explore treatment options, communicate, and promote healing are examples of the art of medicine. Dr. Ventres describes how he has personally worked to cultivate being fully attentive and present by keeping in mind four introspective intentions. These intentions are aimed at alleviating suffering, promoting dignity, recognizing interdependency, and advancing wisdom, respectively. The author presents these intentions, reviews some benefits by which they enhance his work, and discusses concerns. He invites HCPs to consider integrating intentions into their own clinical practices.^1^

Dr. Ofri, an astute observer and prolific medical writer, points out that standardized medicine may mislead HCPs because patients are unique and require creative solutions. She states, “Poetry is one of my favorite tools because of its unselfconscious focus on metaphor. By definition, metaphor requires the stringing together of parts of the mind that don’t normally work together. Master diagnosticians and scientists cogitate in the same way, actively considering ideas that don’t normally sit together… It is our job as clinicians to work with patients to untangle these metaphors. For this, solid medical knowledge is necessary but not nearly sufficient. We need to flex the oddball neurons that connect the disparate corners of our consciousness.”^2^

Another way to think about art and medicine is to consider ***art as medicine***. This may include engagement in art for personal development and maintaining resilience. The *I Medici di McGill* Orchestra, founded in 1989, consists of staff and students of the Faculty of Medicine who offer public concerts throughout the year is one example. This group of doctors/musicians provide a venue for sharing with and creating a coherent group who connect outside clinic settings as well as with the public, at large. Ledger and Joynes^3^ offered a two-week course on Music and Medicine in the UK to medical students; interview data suggested that focusing on music may lead to changes in the way students use music for their own health and well-being.

Reading literature and writing can also serve as a means of personal and professional development. Downie suggests that, “We learn from literature by imaginative identification with the situations or characters in literature, and by having our imaginations stretched through being made to enter into unfamiliar situations or to see points of view other than our own. Learning of this kind is generative of a deep understanding which is essential to humane doctoring.”^4^ Dr. Charon and her colleagues at the College of Physicians and Surgeons of Columbia University teach and promote Narrative Medicine. It is defined as an interdisciplinary field that brings powerful narrative skills of radical listening and creativity from the humanities and the arts to address the needs of all who seek and deliver healthcare. Narrative medicine enables patients *and* caregivers to voice their experiences, to be heard, to be recognized, and to be valued, improving the delivery of healthcare.^5^

Doctors who moon-light as writers are widely read and highly esteemed; famous authors are Chekhov, Williams, Jung, Yalom, Sacks, Gawande, and Ofri and Verghese, among many others. Given physicians recurrent exposure to human drama they can offer insights learned and describe dilemmas confronted in creative ways. Sometimes the stories told can be like a cathartic exercise enabling the clinician to work through emotions that emerged during the therapeutic encounter.^6^ Other authors, such as the surgeon Dr. Selzer, invite readers “behind the scenes” sharing the theatre of mortal existence.^7^ Furthermore, when a doctor’s status changes from healer to patient jarring experiences are described; these differ from patients who have never donned a white coat.^8^ For readers who find this topic interesting, journals that publish essays and poems linking art and medicine are found at https://www.kevinmd.com/blog/2013/03/doctors-publish-literary-writing.html.^9^

Films can effectively communicate a variety of messages relevant to medicine as well. McGill Programs in Whole Person Care presents a “Films that Transform” series each spring that is open to the public. Films are chosen with specific criteria in mind. Following the viewing, a panel consisting of clinicians, film makers and sometimes patients who lead a discussion. Then the public is invited to comment and question the panel members. Medical students, retirees, and anyone curious about healing and medicine may attend.

Hospital corridors sometimes display artwork created by patients. Paintings graphically show how illness experiences have impacted the patient/artist’s life. HCPs may gain perspective regarding what it is like to have chronic pain, breast cancer, or other conditions when they view this work. A virtual gallery of artwork created by patients with chronic pain can be found at http://painexhibit.org/en/.

Art therapy (painting, music) for patients is provided in many hospitals and palliative care units. One example is inviting patients with Parkinson’s Disease (PD) to dance. According to one review, the benefits of dance include improved balance and gait function as well as better quality of life. Most studies of dance for PD have included primarily individuals with mild to moderate PD. While benefits can be obtained with a short, intensive dance intervention, longer interventions may prove to be more effective.^10,11^ A systematic review of art therapy for children (from 2000-2017) with various health problems suggests improvements, but the authors caution that the 13 studies included in the analysis render conclusions premature due to methodological limitations.^12^

Many, if not most, medical schools have recognized the value of including the humanities in the education of future doctors, ***art for medical education***. A person who appreciates art is likely to be open-minded, curious, at ease with the unknown, able to tolerate ambiguity, and to approach artwork with a beginner’s mind – that is as if they were experiencing something for the first time. The need for this is expressed by a third year McGill medical student, Susan M Ge, who wrote, “Due to the technology available to *look into* the patient, doctors have lost the ability to *see* the manifestations of illness from simply observing the external appearance and demeanor of the patient. This void in the area of observation can be filled by the study of art. A piece of artwork holds both the physical and emotional back story of the people depicted in it if one knows how to look.”^13^

How and where to look has been addressed by several medical schools that include guided museum visits^14^ or other forms of art in education (for a review see Mukundaa et al.^15^). Naghshineh et al.^16^ found that students who participated in the “visual literacy” class increased the number of observations made and improved the details of their descriptions, especially those who attended eight or more classes. At the Brigham and Women's Hospital, doctors, nurses, and Harvard medical students use the museum to enhance team building and to break down the hospital hierarchy. Observing and describing art promotes problem solving, communication, thinking creatively, and appreciating other perspectives. While the museum experience aims to interpret artwork together, the goal is to understand a patient’s condition collectively in the clinical setting.^17^

Dr. Pellegrino wrote that engagement in the humanities offers three important benefits to physicians that are essential to their competence: methods of inquiry or thought, content of knowledge, and the power to feed and invigorate the spirit.^18^ One example of a medical school that has taken this to heart is at the University of Iowa Carver College of Medicine – Writing and Humanities program. It advances the connections between the humanities and medicine through the Humanities Distinction Track designed to encourage, support, and recognize medical students who pursue scholarship in creative writing, literature, philosophy, religion, visual arts, music, and history, as well as social sciences and public policy.^19^

In these times of budget cuts and streamlining medical services, I would caution against leaving humanities out of training and continuing personal and professional development for physicians and other HCPs. By allowing art to enhance their well-being and contribute to comprehensive care we can be supportive of their efforts to become the type of physician they aspire to be. Moreover, clinicians may come to understand how art can increase their patients’ quality of life.
